# Effect of Reticulate Unit Spacing on Microstructure and Properties of Biomimetic 7075 Aluminum Alloy by Laser Cladding

**DOI:** 10.3390/mi14020418

**Published:** 2023-02-10

**Authors:** Qi Sui, Danyang Cheng, Yingfei Dong, Yijia Ma, Yingrui Su, Ning Hu, Zexuan Sun, Yuanbo Chen

**Affiliations:** Key Laboratory of Advanced Structural Materials, Ministry of Education, School of Materials Science and Engineering, Changchun University of Technology, Changchun 130012, China

**Keywords:** 7075 aluminum alloy, laser cladding, biomimetic surface, strength and toughness

## Abstract

In the context of energy conservation and emission reduction, more and more attention has been paid to the development of lightweight metal materials with both high strength and high toughness. Inspired by the non-smooth surface of natural organisms, a biomimetic surface with various spacing reticulate units of 7075 aluminum alloys was modified by laser cladding. The microstructure, microhardness and tensile properties of the various spacing units with CeO2-SiC-Ni60 were studied. The finer microstructure and the higher microhardness of various spacing units in comparison with that of 7075 aluminum alloys were obtained, no matter the strip-like treated region or the cross-junction region. Moreover, the best combination of strength and toughness of the biomimetic sample with 2.5 mm spacing reticulate unit was discussed. Finally, by combining the microstructure, XRD phase change, thermal gradient effect, thermal expansion coefficient difference and hard phase strengthening mechanism, it was concluded that the 2.5 mm spacing reticulate unit had the best ability to inhibit crack propagation, and the dispersive hard phases of Al_3_Ni_2_ and SiC played a major role in stress release of the matrix.

## 1. Introduction

Since modern times, with the rapid development of human industrialization, energy conservation and emission reduction has become a key point that has to be paid attention to in current social development and technological progress [1]. In this context, the application of lightweight materials has become increasingly prominent [2]. Because of its unique mechanical properties and excellent processing effect, 7075 aluminum alloy has been widely used in the aerospace industry, molds, fixtures and other fields [3,4]. With the continuous maturity of current industrial technology and the increasing emphasis on environmental protection, researchers have conducted many investigations into the modification of new lightweight 7075 aluminum alloys [5].

In the strength-toughening process of light metal materials such as 7075 aluminum alloy [6], laser technology [7,8] has attracted a wide range of applications due to its low dilution rate, fast speed, flexibility and greater saving of raw materials [9,10]. Yuxin Li et al. [11] used laser cladding technology to prepare Ti/TiBCN coating on the surface of 7075 aluminum alloy. The results show that when the TiBCN content is 15%, the average hardness of the Ti/TiBCN cladding coating was 750 HV_0.2_, which was about five times higher than that of the substrate (~127 HV_0.2_), and the coating mass loss was only 2.4 mg in 20 min. T. M. Yue et al. [12] used the laser double melting technique to study the wear properties of Al_2_O_3_ bearing coatings prepared via in situ laser-induced thermite reaction on 7075 aluminum alloy. It was finally found that under dry sliding conditions, the wear resistance of the laser clad samples was much higher than that of the unclad samples.

**Figure 1:**
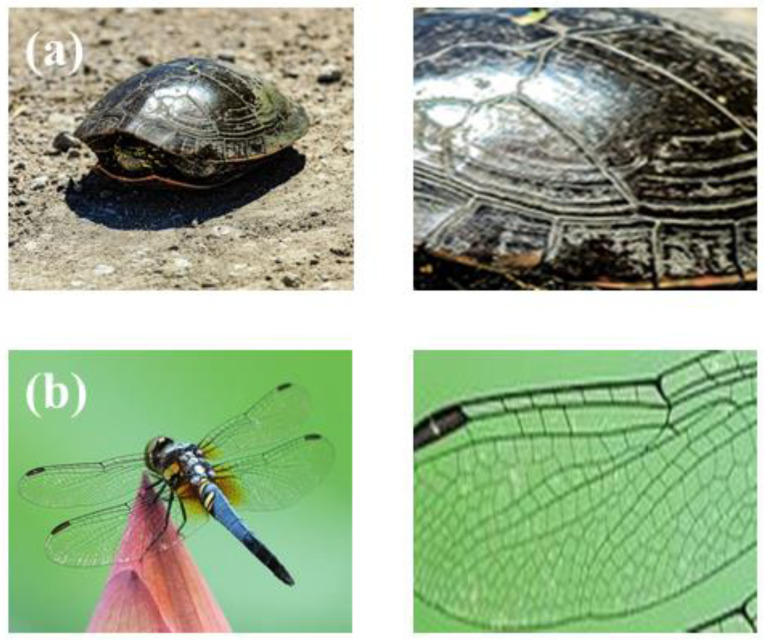
Biomimetic structures in living organisms and their partial magnifications: (**a**) turtle; (**b**) dragonfly.

In addition, biomimetic science, inspired by the various unique structures of natural organisms that have evolved over millions of years and formed to adapt to the living environment, has played a very effective role in the design and processing of materials [13,14]. Inspired by the non-smooth surface of animals, Yuhuan Yuan et al. [15] used laser remelting technology to fabricate biomimetic units of various shapes on the surface of 7075 aluminum alloy. Through the methods of microscopic characterization, wear performance testing and finite element analysis, it was finally concluded that the wear resistance of the samples with biomimetic shapes was significantly improved compared with the unprocessed samples. Further observation of the high-strength and high-flexibility parts of animals and plants shows that it is a reticulation-like structure with “soft-hard” spacings [16,17]. Figure 1 shows turtle shells and dragonfly wings with a “soft-hard” reticulation-like structure.

From the above, we can clearly observe that the current research on the modification of 7075 aluminum alloy via laser technology focuses on the improvement of strength, while research on the influence of toughness and its mechanism while the strength is increased is very rare. 

Therefore, in this study, the surface of 7075 aluminum alloys were modified via laser cladding mixed powder of SiC ceramic particles and rare earth compound CeO_2_. Moreover, inspired by the non-smooth biomimetic structure, the reticulation-like units with “soft-hard” intervals were processed. By means of XRD, SEM, micro-hardness and tensile property testing, the microscopic mechanism of the matrix and processing units on the toughness of the sample was explored.

## 2. Experimental Materials and Methods

### 2.1. Materials

The base material was 7075 Al alloy, and its chemical composition was shown in Table 1.

The cladding material was a mixed powder composed of Ni_60_, SiC ceramic particles and rare earth mixture CeO_2_. Among them, the Ni-based self-fluxing alloy powder came from MCC Xindun Alloy, China, and its particle size ranges from 50 to 82 μm. Moreover, both SiC and CeO_2_ powders were obtained from Hebei Bocheng Metallurgical Research Center (China), and their specifications were in the range 20–50 μm and 2–9 μm, respectively.

### 2.2. Methods

#### 2.2.1. Sample Preparation

The matrix material 7075 Al alloy was processed into a sample with a size of 72.6 mm × 16 mm × 3 mm by Huadong Dk7732 CNC wire EDM, so as to observe the macroscopic morphology and microstructure. Before the laser processing of the biomimetic sample, the Al_2_O_3_ film, oil stains and dirt on the surface of the 7075 aluminum alloy material were removed via a mixed method of physics and chemistry. First, #800, #1000 and #1200 water sandpapers were used to grind and polish the substrate continuously to ensure the smooth surface of the sample to be treated. Then, ultrasonic cleaner, acetone and alcohol were used for cleaning. In addition, the cladding material was a mixed powder of Ni-based + 15% SiC + 3% CeO_2_. Moreover, in order to ensure the uniformity of the processing molten pool to the greatest extent, the mixed powder was ball-milled in a planetary ball mill (QM-3SP2) with a ball-to-material ratio of 2:1 and a rotational speed of 400 r/min for 36 h. The mixed powder and water glass are thoroughly and evenly stirred in proportion, spread on the surface of the sample with plastic wrap, and compacted, air-dried, ground to uniform thickness and ultrasonic cleaned in turn.

**Figure 2:**
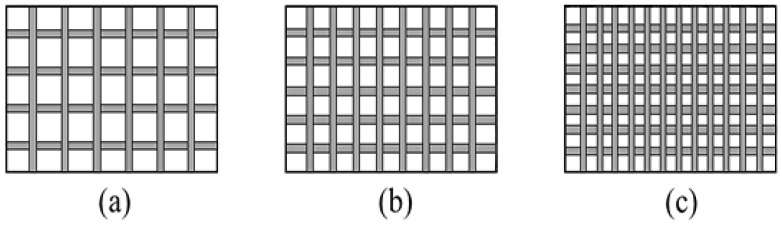
Schematic diagram of the processing of the biomimetic sample: (**a**) 3.5 mm; (**b**) 2.5 mm; (**c**) 1.5 mm.

The laser used was a 20 kW ZGCM series pulsed laser heat treatment complete set of equipment. It is large-scale laser heat treatment equipment with high intelligence and good precision produced by Wuhan Chutian Industrial Laser Equipment Co., Ltd. The processing system is composed of a three-dimensional movable mechanical spray gun, a workbench, a cooling cabinet, a protective gas cylinder for falling gas and a digital control system. Among them, under argon as the protective gas, a defocus value of 11 mm, the current of 140 A, a laser frequency of 5 Hz, a pulse width of 5 ms and a scan rate of 60 mm/min were selected. The biomimetic samples were prepared by designing different unit spacings, where the center distances of the machining units were 1.5, 2.5 and 3.5 mm, respectively. The schematic diagram of the processed samples is illustrated in Figure 2.

#### 2.2.2. Performance Characterization

**Figure 3:**
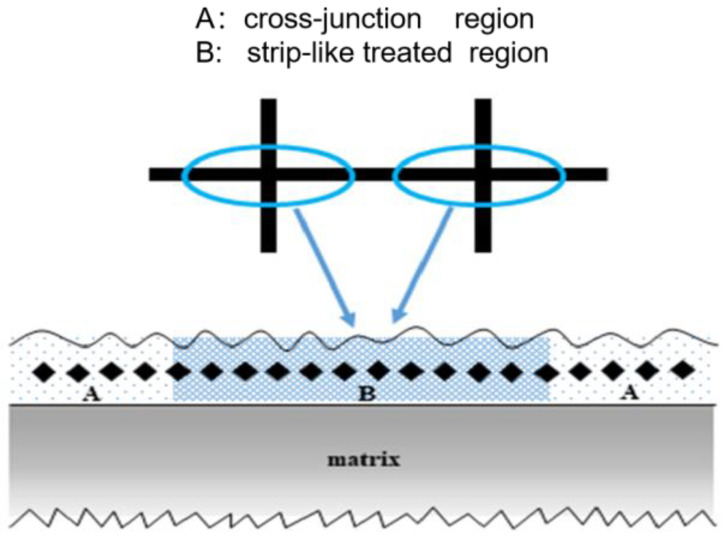
Schematic diagram of microhardness measurement in areas A and B in the vertical direction of laser processing.

The macro-morphology, microstructure, phase composition and average microhardness of the biomimetic samples were investigated. Tensile properties were tested and microscopic mechanisms were analyzed. After grinding, polishing and alcohol cleaning of all samples, the macroscopic morphology of the molten pool at the cross-section of the biomimetic samples along the vertical direction of laser processing was observed under a research-grade inverted metallographic microscope of DM13000H after being corroded with Keller reagent. The microstructures at the strip-like treated region A and at the cross-junction region B of the processed samples were observed using a field emission scanning electron microscope (Zeiss, Gemini Supra 40, Oberkochen, Germany). The changed in phase composition of the samples before and after processing were observed via an X-ray diffractometer (Rigaku D/max 2500, Tokyo, Japan) operated under Cu Ka radiation, voltage of 40 kV, current of 40 mA and scanning speed of 1°/min. The average microhardness at regions A and B obtained from five measurements was measured using a Vickers microhardness tester (Buehler, 1600-5122VD MicroMet 5104, Lake Bluff, IL, USA), where a load size of 25 gf and a load time of 15 s were selected, and the relevant schematic diagram of the measurement is presented in Figure 3.

**Figure 4:**
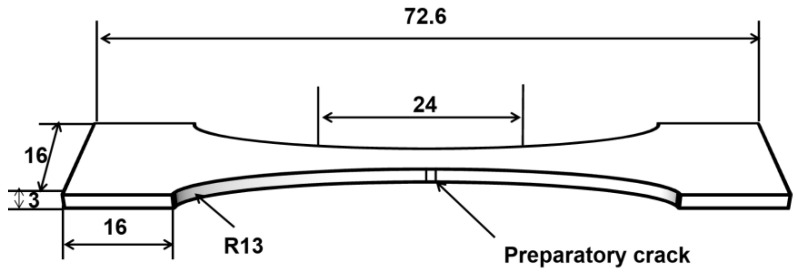
Dimensions of the tensile sample.

**Figure 5:**
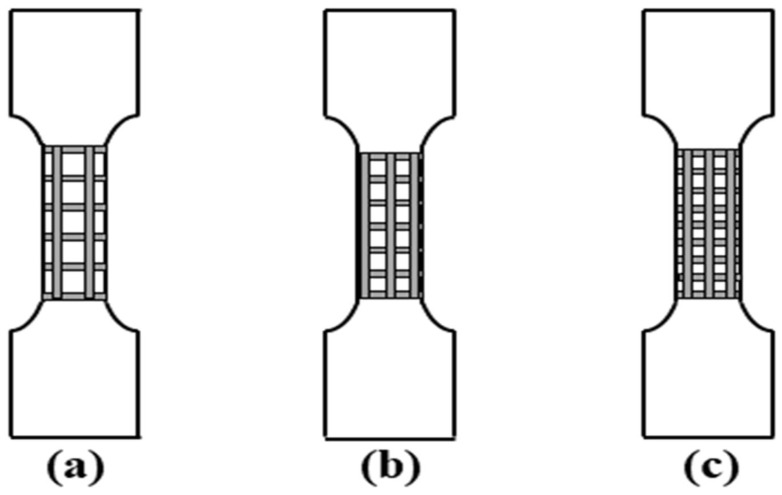
Processing diagram of biomimetic tensile samples with different spacings: (**a**) 3.5 mm; (**b**) 2.5 mm; (**c**) 1.5 mm.

The tensile properties of the biomimetic samples were tested in accordance with the Metal Materials-Tensile Test-Room Temperature method (GB/T 228.1-2010) and using a WDW-100 micro-controlled electronic universal testing machine. In order to more intuitively analyze the crack propagation of the samples during the tensile process, the tensile samples were pre-cracked in this study, and the shape, size and location of the pre-cracks are presented in Figure 4 (the unit of data is mm). The schematic diagram of the tensile sample processing combined with the biomimetic reticulate design is shown in Figure 5, and in order to ensure the stability of the chuck during the test, only the working area was processed in this study. During the test, a room temperature of 24 °C and a strain rate value of 3.5 × 10^−4^ s^−1^ were chosen, and all values were taken from the average of five measurements under the same conditions. In addition to the values obtained from the test, SEM was used to observe the tensile fracture surface at the pre-crack, and to explore the microscopic mechanism of the influence of the biomimetic structure on its performance.

## 3. Results and Discussion

### 3.1. Microstructures

**Figure 6:**
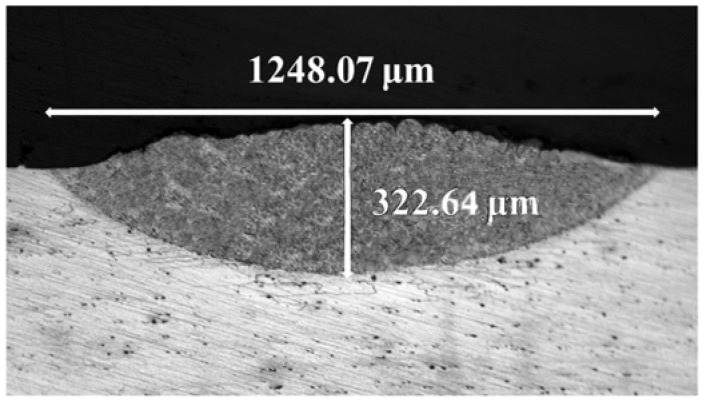
The macroscopic cross-sections of the units.

Figure 6 shows the morphology of the cross-section of the processed region (unit) after grinding, polishing and corrosion with Keller reagent. The width and depth of the unit were 1248.07 μm and 322.64 μm, respectively. The white light part is the matrix and the dark parabola part shows the unit. Due to the influence of the heat-affected zone, the temperature in the molten pool showed a gradient change, and the energy absorption degree of each part was different [18].

**Figure 7:**
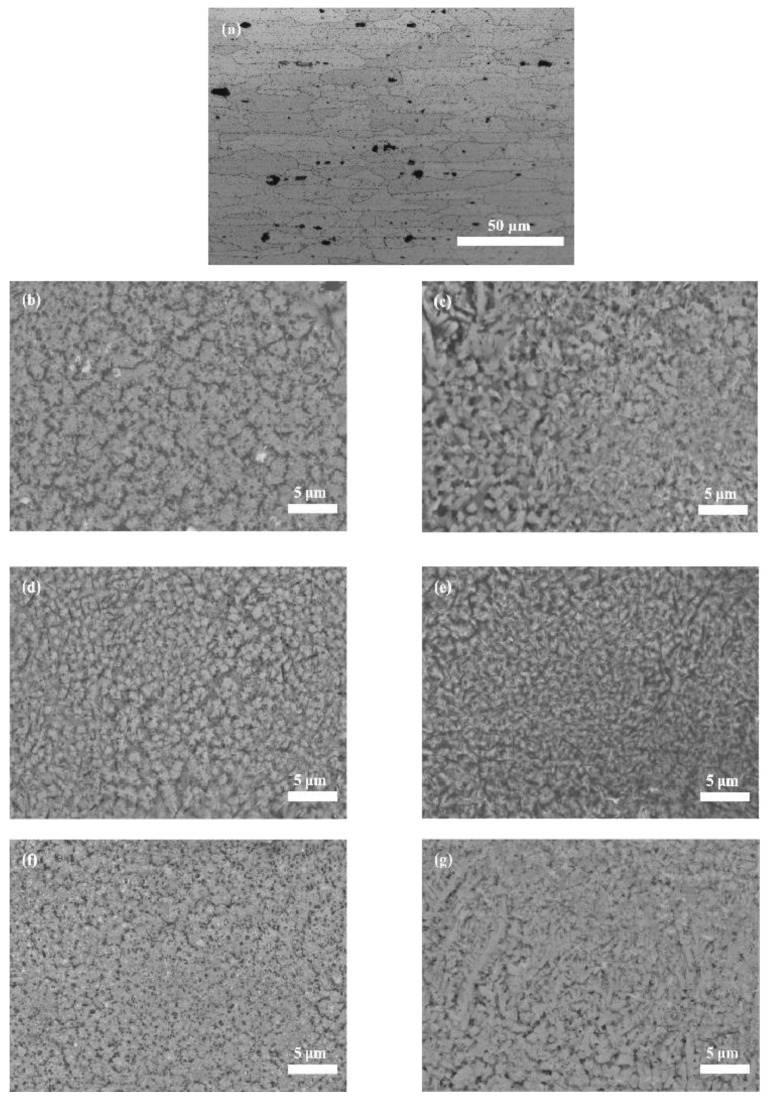
SEM microstructure at regions A and B of the biomimetic samples with different spacing: (**a**) matrix, (**b**) 1.5 mm A, (**c**) 1.5 mm B, (**d**) 2.5 mm A, (**e**) 2.5 mm B, (**f**) 3.5 mm A, (**g**) 3.5 mm B.

The microstructure of the matrix and units is shown in Figure 7. Figure 7a shows the microstructure of the unprocessed sample. Figure 7b,d,f show the microstructures of the strip-like treated region A with spacings of 1.5, 2.5 and 3.5 mm, respectively. By comparing the microstructures at region A with the matrix, it was easy to find that the crystal grains were significantly refined. This was because region A melts and solidifies at an instantaneous speed in the laser cladding area, resulting in an extremely fast cooling rate and a fine grain structure. Further analysis of the microstructure of region A under different biomimetic spacings showed that the grain refinement effect of region A gradually deteriorates with the reduction of laser biomimetic spacings. This was because during the solidification process, the ratio of temperature gradient to solidification rate controls the crystallization growth morphology, resulting in an uneven distribution at the units with large spacing, as shown in Figure 7b,d,f. Furthermore, with the increase of spacing, the less affected it is by the processing temperature of adjacent unit, the slower the growth rate of grains in the molten pool, and there is a tendency to grow unevenly, resulting in slow melting and uneven grain growth, as shown in Figure 7f. Therefore, combining the technical characteristics of fast melting and quenching in laser processing and the comparative analysis of the microstructure can predict that the optimal spacing for laser cladding biomimetic reticulation processing was 2.5 mm.

In addition, the microstructure morphology at the cross-junction region B of samples processed with different unit spacings of 1.5, 2.5 and 3.5 mm is also shown in Figure 7c,e,g, respectively. Compared with region A, the effect of the change of spacing on the coarsening and uneven distribution of grains in region B is less obvious, which is mainly due to the fact that the laser processing at region B was equivalent to another advanced technology in laser surface modification processing—laser multilayer cladding technology. In the process of two huge temperature differences, more crystal nuclei are formed, so that the grain growth space is extremely limited, so that region B’s organization is more dense and uniform than region A’s organization, as shown in Figure 7b–e or Figure 7f,g. It is noteworthy that it can be found that different degrees of fine dendrite microstructure appear at region B by comparing with that of the region A. Further, through the distribution law of the temperature field in the multilayer cladding [19], it can be concluded that the appearance of fine dendrites was due to the formation of a large temperature gradient field at the interface during the second layer cladding process, coupled with the large super-cooling effect of the material itself and its high thermal conductivity, fine dendrite in region B formed a part, which play a positive role in strengthening the surface of the substrate [20,21].

In summary, the biomimetic surface of 7075 aluminum alloy with 2.5 mm spacing could be used to obtain relatively uniform and excellent fine microstructure at both regions A and B, thus contributing to the improvement of the overall performance of the sample.

**Figure 8:**
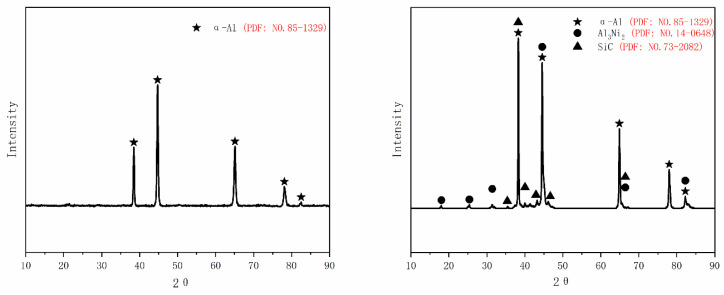
XRD patterns of the substrate and the biomimetic sample.

The phase analysis spectrum of the matrix and the biomimetic sample is shown in Figure 8. Clearly, phase compositions of the matrix and the biomimetic sample mainly include α-Al according to PDF No.85-1329. Moreover, in addition to the SiC and α-Al, compounds formed via Ni, SiC and Al matrix also appeared on the surface of the cladding layer. By comparing the standard PDF cards with serial numbers No.14-0648 and No.35-0799, it was analyzed that Al_3_Ni_2_ was produced in the cladding layer. During the solidification process of the molten pool, according to the Al-Ni phase diagram, it can be seen that two phase change reactions of reaction Formulas (1) and (2) mainly occurred.

(1) L→NiAl

(2) $$L+\mathrm{NiAl}\overset{1300\;℃}{\to }{\mathrm{Al}}_{3}{\mathrm{Ni}}_{2}$$

Both Al_3_Ni_2_ and SiC were hard phases with high hardness, and as the second phase in the cladding layer, Al_3_Ni_2_ played a role of strengthening the second phase. It was predicted that the existence of the hard phase will restrain the crack propagation to a certain extent when the sample was fractured or failed under stress.

### 3.2. Microhardness

**Figure 9:**
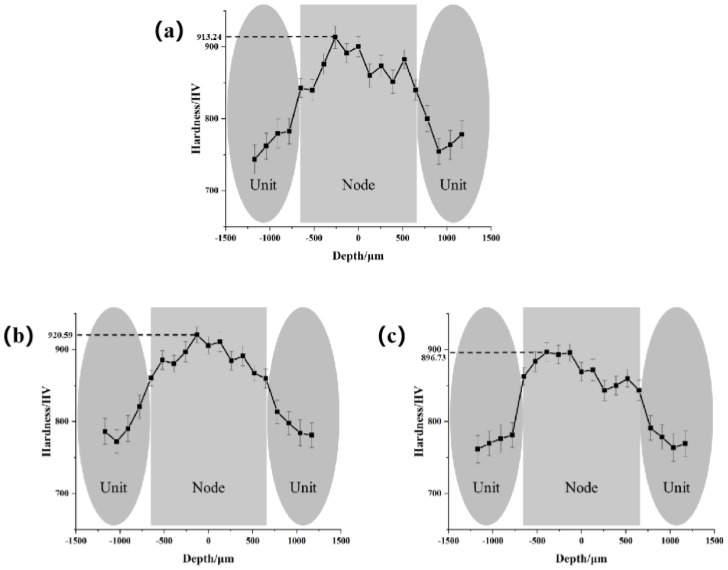
Vickers Microhardness at regions A and B: (**a**) 1.5 mm, (**b**) 2.5 mm, (**c**) 3.5 mm.

In general, hardness reflects the plasticity resistance of a material, and hardness was proportional to strength [22]. In previous studies, the average microhardness of the 7075 aluminum alloy matrix was $180_{-6}^{+9}$ HV_0.2_. However, from the microhardness distribution near the biomimetic cross-junction regions in Figure 9, it could be clearly seen that the change at region A was from 743.56 to 820.57 HV_0.2_, which was 313% to 356% higher than that of the matrix, and the change at region B was from 839.75 to 920.59 HV_0.2_, which was a further 53% increase compared with region A. Moreover, the microhardness of region A decreased as the distance from region B increased, which was caused by the gradual weakening of the thermal influence as the laser center point expands during the reticulation processing. In addition, the microhardness of region B of the biomimetic samples was higher than that of region A to varying degrees. Among them, the region B microhardness of 2.5 mm reticulation was as high as 920.59 HV_0.2_ and the value was more uniform, which was higher than that of 1.5 mm and 3.5 mm, so it could be inferred that the spacing with 2.5 mm was the best value for strength improvement processing.

### 3.3. Tensile Results of Biomimetic Samples with Different Spacings

**Figure 10:**
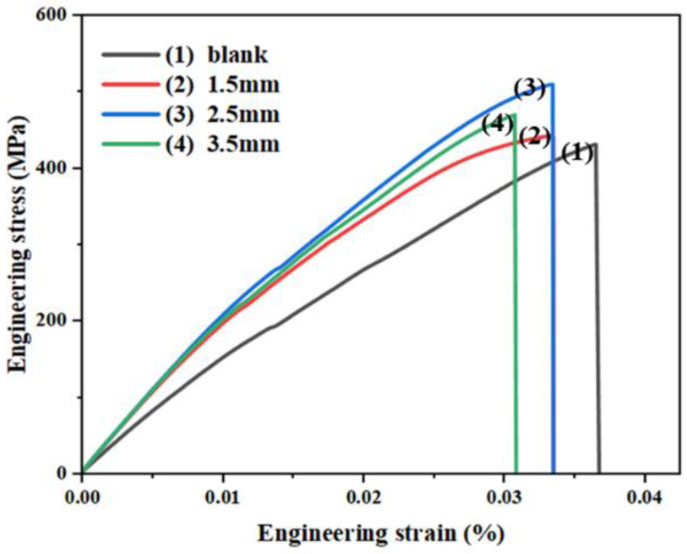
Tensile stress–strain curves of different samples.

Figure 10 presents the stress–strain curves of the unprocessed and processed samples with various spacing units. The ultimate tensile strength of all processed samples was superior to that of the unprocessed samples with little reduction in elongation at break. Further compared with the samples with 1.5 and 3.5 mm spacings, the samples with 2.5 mm spacing showed excellent tensile properties. As can be seen from Figure 7, the unit spacing with 2.5 mm could make the microstructure of both regions A and B more uniform and refined, which was beneficial to the suppression of crack initiation and propagation in the tensile process, resulting in excellent tensile properties. In addition, it could also be observed that samples with 1.5 mm spacing exhibit poor tensile properties. This was caused by the fact that more cladding powder would be added to the sample surface with the decrease in the spacing. The cladding powder also contained hard SiC ceramic particles, which increased the brittleness of the sample. Due to the “slotted” design of the standard tensile sample, there was a stress concentration phenomenon during the stretching process; the yield process cannot be visually observed in the stress-strain curve. In addition, Table 2 lists the average values of the ultimate tensile strength (UTS) and the elongation (EL) calculated from Figure 10. When the spacing increased, the UTS value increased first and then decreased, while the EL value changed slightly due to stress concentration. In particular, the sample reached the highest UTS of 510 MPa and an EL of 0.033% when the unit spacing was 2.5 mm. The UTS increased by 15% compared with the lowest processed sample of 1.5 mm at 443 MPa; moreover, the elongation at break decreased by only 11% compared to the blank sample.

### 3.4. Fracture Surface

**Figure 11:**
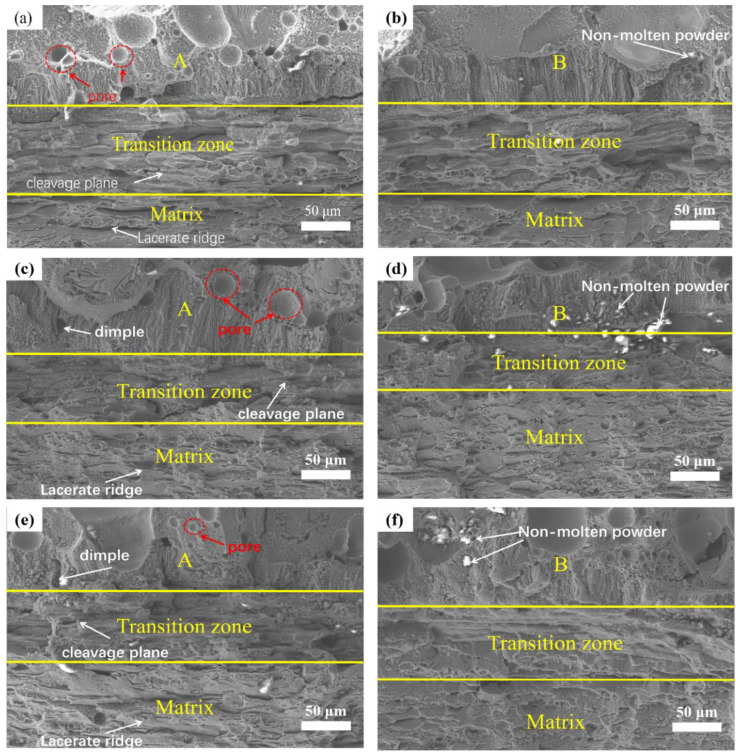
The fracture surfaces of the samples with different biomimetic spacings in regions A and B: 1.5 mm (**a**,**b**), 2.5 mm (**c**,**d**) and 3.5 mm (**e**,**f**).

Figure 11 shows the fracture surfaces of samples with different spacings at regions A and B, respectively. Obviously, biomimetic samples with different spacing units showed distinct differences in fracture surface morphology in the laser processing zone (regions A and B), transition zone and matrix. For the matrix, it could be observed that the fracture surface was mainly composed of tearing edges. By comparing the tearing edge with the grain size of the matrix (Figure 7a), it was found that their average size was 60 μm, suggesting that inter-granular fracture occurred during the fracture process. By comparing the fracture surfaces of biomimetic samples with different spacings (Figure 11a,c,e or Figure 11b,d,f), it could be clearly observed that the fracture surfaces of samples with 2.5 mm spacing were more uniform and smaller than those of other samples, no matter the tearing edge at the matrix or the cleavage step at the transition region. This indicated that under the action of tensile force, the biomimetic sample with 2.5 mm spacing unit exhibited better tensile fracture resistance, which corresponded to the stress–strain curve in Figure 10 and the data calculated in Table 2. In addition, according to the morphology analysis of the fracture surface at regions A and B with the same spacing (Figure 11a,b or Figure 11c,d or Figure 11e,f), the fracture surface at region B presented finer and more uniform morphology than that at region A, which also indicated that region B had a better anti-crack effect in the tensile process, corresponding to the microstructure analysis in Figure 7.

**Figure 12:**
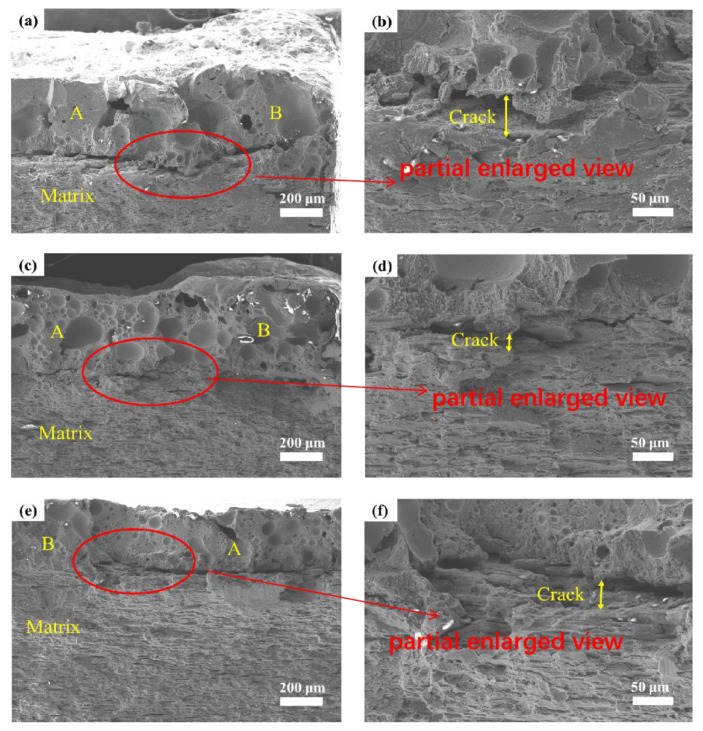
The fracture surfaces at the junction of regions A and B (**a**,**c**,**e**), and their partial enlarged view (**b**,**d**,**f**).

When observing and analyzing the fracture surfaces at regions A and B of samples with different unit spacings, it could also be found that micro cracks of different degrees occurred at the junction of regions A and B of different samples during the loading process of tensile force, as shown in Figure 12. The formation of cracks at the junction reflected the slow release of the stress of the biomimetic samples during the loading process. The larger the crack size, the more significant the stress concentration and the greater the fracture damage. By comparing the length and width of the crack at the junction, it was found that the crack of the sample with the spacing of 1.5 mm (Figure 12a,b) was the most serious in both the transverse and longitudinal direction, and even the width reached the maximum 53 μm. In contrast, for the 2.5 mm sample, after loading, the crack was extremely small compared to the 1.5 mm sample, with a width of 20 μm, which was about 60% improvement. The phenomenon showed that the “soft-hard” interphase structure had the best stress-releasing effect in the processing of biomimetic samples with a spacing of 2.5 mm reticulate unit; that is to say, the damage between the unit and the matrix reached the maximum value during the loading process, which increased the tensile properties of the sample.

### 3.5. Mechanism

The high-hardness SiC ceramic particles increased the brittleness of the material. This was caused by the great difference in thermal expansion coefficient between ceramic particles and metal matrix (SiC = 3.8 ppm/k [23] and Al = 24.2 ppm/k [23]); some ill-bonded areas existed as crack sources during sample processing and loading process, which negatively affected the mechanical properties of materials. The change of the unit spacing directly affected the SiC content on the surface of the biomimetic sample. During the tensile test, as stress was transferred from the softer base to the harder units, the harder units were likely to concentrate the entire tensile stress. Therefore, as the SiC content was large (the biomimetic spacing was 1.5 mm), almost all the stress was concentrated in the cladding layer, which had adverse effects on the combination of the cladding layer and the matrix, and was not conducive to the original design of the “soft-hard” interphase structure, hoping that the “soft” material had a certain slow-release effect on the stress. Similarly, as the SiC content was low (the unit spacing was 3.5 mm), the processed area could not bear more stress during the tensile process. When the appropriate content of SiC was added, the cladding layer had higher stress, while the matrix had lower stress. Therefore, it was necessary to apply greater external force to conquer the laser processing area, and the ultimate tensile strength of the biomimetic sample was increased.

**Figure 13:**
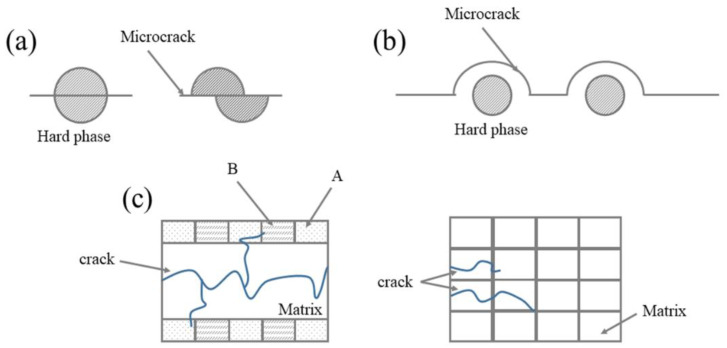
Schematic diagram of the hindrance of hard phase and biomimetic structure with respect to crack propagation: (**a**) Microcrack shear through the hard phase; (**b**) Microcracks bypass the hard phase; (**c**) The biomimetic reticulation structure hinders the propagation of cracks in the longitudinal and transverse directions.

In addition to withstanding greater stress, the existence of SiC and Al_3_Ni_2_, hard phases had an impeding effect on the development of micro-cracks caused by stretching, which could not be ignored. When the crack expanded from the matrix to the laser-processed region, the crack had to consume some energy to bypass or shear through the hard phase dispersed at regions A or B, which hindered the crack propagation (as shown in Figure 13a,b). In other words, more external force was required to make the crack continue to expand and fracture occurred. In addition, the effect of reticulate region B on crack propagation was similar to that of hard phase (as shown in Figure 13c).

On the other hand, the difference in unit spacings would affect the temperature gradient in the heat-affected zone of the sample. Too large or too small temperature gradient was not conducive to the formation of the optimal microstructure of processing area. The uniformity and refinement of microstructure played a key role in the mechanical properties of the whole material, which would make the material have less crack initiation during the loading process.

## 4. Conclusions

This paper addressed biomimetic samples with reticulate units (CeO_2_-SiC-Ni_60_) composed of strip-like regions and the cross-junction regions with different spacings which were prepared on the substrate of 7075 aluminum alloys by laser cladding. Through the analysis of structure, phase change, microhardness, tensile test and microscopic mechanism, the following main conclusions were drawn:

(1) Compared with the unprocessed region of biomimetic samples, the microstructure of the processed regions (units) with various spacings were finer, and the microhardness was improved. Moreover, spacings of reticulate unit affected the microstructure and microhardness. Among the microstructure of the processed regions (units) with various spacings, compared to 1.5 mm and 3.5 mm, 2.5 mm had the finest crystal and the highest microhardness, no matter the strip-like region or the cross-junction region.
(2) Among the microstructure of the processed regions (units) with various spacings, compared to 1.5 mm and 3.5 mm, the strength and tensile properties of the biomimetic samples with 2.5 mm spacing were the best, and the UTS increased by 15% compared with the lowest processed sample of 1.5 mm.
(3) Among the microstructure of the processed regions (units) with various spacings, compared to 1.5 mm and 3.5 mm, the “soft-hard” interphase structure in the 2.5 mm spacing reticulation design played an excellent role in slowing down the stress on the matrix, to avoid stress concentrated in low strength areas during the loading process, and the fracture damage between the unit and the matrix was minimal. In addition, the cross-junction region of the reticulate unit played a major role in inhibiting the initiation and the growth of cracks; the ability mainly depended on the uniformity of the microstructure and the distribution of the Al_3_Ni_2_ hard phases.

## Figures and Tables

**Table 1:** Composition of 7075 Al alloy (wt.%).

Component	Zn	Mg	Cu	Cr	Fe	Si	Mn	Ti	Al
**Content**	5.53	2.63	1.51	0.22	0.16	0.06	0.04	0.03	Bal

**Table 2:** Tensile property test results with different unit spacings.

Sample	UTS, MPa	EL, %
blank	$432_{-5}^{+3}$	$0.037_{-0.001}^{+0.002}$
1.5 mm	$443_{-8}^{+4}$	$0.033_{-0.001}^{+0.001}$
2.5 mm	$510_{-7}^{+6}$	$0.033_{-0.001}^{+0.002}$
3.5 mm	$470_{-10}^{+4}$	$0.031_{-0.002}^{+0.001}$

## Data Availability

Not applicable.
